# A systematic review and meta-analysis of the diagnostic accuracy of metagenomic next-generation sequencing for diagnosing tuberculous meningitis

**DOI:** 10.3389/fimmu.2023.1223675

**Published:** 2023-09-26

**Authors:** Zheng-Bing Xiang, Er-Ling Leng, Wen-Feng Cao, Shi-Min Liu, Yong-Liang Zhou, Chao-Qun Luo, Fan Hu, An Wen

**Affiliations:** ^1^ Department of Neurology, Jiangxi Provincial People’s Hospital (The First Affiliated Hospital of Nanchang Medical College), Nanchang, Jiangxi, China; ^2^ Department of Neurology, Xiangya Hospital, Central South University, Jiangxi Hospital, National Regional Center for Neurological Diseases, Nanchang, Jiangxi, China; ^3^ Department of Pediatrics, Jiangxi Provincial People’s Hospital (The First Affiliated Hospital of Nanchang Medical College), Nanchang, Jiangxi, China

**Keywords:** tuberculous meningitis, cerebrospinal fluid, metagenomic next-generation sequencing, diagnosis, meta-analysis

## Abstract

**Objective:**

The utility of metagenomic next-generation sequencing (mNGS) in the diagnosis of tuberculous meningitis (TBM) remains uncertain. We performed a meta-analysis to comprehensively evaluate its diagnostic accuracy for the early diagnosis of TBM.

**Methods:**

English (PubMed, Medline, Web of Science, Cochrane Library, and Embase) and Chinese (CNKI, Wanfang, and CBM) databases were searched for relevant studies assessing the diagnostic accuracy of mNGS for TBM. Review Manager was used to evaluate the quality of the included studies, and Stata was used to perform the statistical analysis.

**Results:**

Of 495 relevant articles retrieved, eight studies involving 693 participants (348 with and 345 without TBM) met the inclusion criteria and were included in the meta-analysis. The pooled sensitivity, specificity, positive likelihood ratio, negative likelihood ratio, diagnostic odds ratio, and area under the summary receiver-operating characteristic curve of mNGS for diagnosing TBM were 62% (95% confidence interval [CI]: 0.46–0.76), 99% (95% CI: 0.94–1.00), 139.08 (95% CI: 8.54–2266), 0.38 (95% CI: 0.25–0.58), 364.89 (95% CI: 18.39–7239), and 0.97 (95% CI: 0.95–0.98), respectively.

**Conclusions:**

mNGS showed good specificity but moderate sensitivity; therefore, a more sensitive test should be developed to assist in the diagnosis of TBM.

## 1 Introduction

Tuberculous meningitis (TBM) is a type of central nervous system (CNS) infection caused by *Mycobacterium tuberculosis* (MTB) invading meninges and spinal membranes. TBM is associated with high disability and mortality rates (1). It is the most severe type of extrapulmonary tuberculosis (EPTB) with the worst prognosis, and is a serious health threat causing a high economic burden to society worldwide (2–4). The onset of TBM is often insidious and its clinical manifestations have variable severity and lack specificity (5). Diagnosis of TBM depends on the detection of MTB in the cerebrospinal fluid (CSF) (6). Owing to the low load of MTB in the CSF, the detection rate of conventional MTB assays is unsatisfactory, leading to difficulty in making an early microbiological diagnosis (7).

Timely and accurate diagnosis at an early stage and initiation of antituberculosis therapy is key to improving the survival rate and prognosis of patients with TBM (8, 9). Recently, attention has been paid to the role of molecular diagnostics in precision diagnosis and treatment. Among them, metagenomic next-generation sequencing (mNGS) is a revolutionary technology that has emerged in recent years and can be used to conduct high-throughput sequencing of microbial nucleic acid in clinical samples and identify pathogens through comparison and analysis with standard sequences in the database (10, 11). This technique has played an increasingly important clinical role in the diagnosis of CNS infections (12–14).

A literature search revealed that no systematic quantitative analysis has been conducted of current studies on the use of mNGS in the diagnosis of TBM. Most studies of the diagnostic accuracy of mNGS for diagnosing TBM are case-control studies with a small sample size, and the reported sensitivity varies greatly; therefore the diagnostic value of mNGS for diagnosing TBM is still unclear. Hence, we conducted a systematic literature review and meta-analysis to systematically and objectively assess the value of mNGS in the diagnosis of TBM.

## 2 Methods

### 2.1 Search strategy

We conducted a comprehensive computerized search of English (PubMed, Medline, Web of Science, Cochrane Library, and Embase), and Chinese (CNKI, Wanfang, and CBM) databases for studies on the use of mNGS for TBM diagnosis. We also manually searched the list of included references to identify additional relevant studies. The target keywords that we used were various combinations of “tuberculosis”, “TB”, “*Mycobacterium*”, “MTB”, “tuberculous meningitis”, “TBM”, “extrapulmonary tuberculosis”, “EPTB”, “cerebrospinal fluid”, “CSF”, “metagenomic next-generation sequencing”, “mNGS”, “next-generation sequencing”, “accuracy”, “sensitivity”, and “specificity”. The retrieval period was from the establishment of each database until February 1, 2023.

### 2.2 Study selection

Studies were included if they met all the following criteria:

(i) Published prospective or retrospective studies of mNGS technique for diagnosing TBM;
(ii) Reference standard for the diagnosis of TBM (test group) was acid-fast staining, culture, or nucleic acid amplification tests (NAATs) of the CSF, or a composite reference standard (CRS);
(iii) The control group included patients with diseases that are clinically confused with TBM, including suppurative meningitis, fungal meningitis, viral meningitis, and other CNS infections;
(iv) The specimen used for mNGS was CSF;
(v) Access to the full text.

Studies were excluded if they met any of the following criteria:

(i) Duplicate studies;
(ii) No non-TBM control group was included, or the control group included only healthy individuals;
(iii) The research question was inconsistent with that of this study;
(iv) Animal experiments;
(v) Reviews, systematic reviews, or meta-analyses;
(vi) Case reports, abstracts, conference abstracts, comments, or letters;
(vii) The 2×2 table data could not be extracted directly or indirectly.

### 2.3 Data extraction

Two investigators independently conducted literature searches according to pre-established criteria, and screening was conducted according to the inclusion and exclusion criteria. The data extracted from the included studies included the author, publication year, study site, study design (prospective or retrospective), age of study participants, reference standard, sample size, sample condition (fresh or frozen), patient selection method, pretreatment of CSF specimens, and 2×2 table data. For the screening of the above studies and the extraction of relevant materials and data, any discrepancies were resolved through open discussion and consultation with a third researcher.

### 2.4 Quality assessment

The quality of the studies was independently assessed by two reviewers using Review Manager software (version 5.3). Quality Assessment of Diagnostic Accuracy Studies-2 (QUADAS-2) was used to evaluate the risk of bias and applicability concerns of all the included studies (15), and a literature quality evaluation chart was drawn.

### 2.5 Statistical analysis

Statistical analyses were performed using STATA 15.0, and *P* values < 0.05 were considered to be statistically significant. The pooled sensitivity, specificity, positive likelihood ratio (PLR), negative likelihood ratio (NLR), and diagnostic odds ratio (DOR) were calculated for the included studies with 95% confidence intervals (CIs). Cochran’s Q test was used to test heterogeneity among the selected studies, and heterogeneity was measured by *I*-square (*I* ^2^) statistics. The appropriate statistical analysis model was selected for the meta-analysis based on the heterogeneity test results. A summary receiver-operating characteristic (SROC) curve was drawn and the area under the curve (AUC) was calculated. Deeks’ funnel plot was drawn to detect publication bias (16), with the level of statistical significance set at α = 0.05. To evaluate the role of mNGS in the diagnosis of TBM, Fagan’s nomogram was used to compare pre- and post-test probabilities.

## 3 Results

### 3.1 Literature search results and the characteristics of the included studies

**Figure 1:**
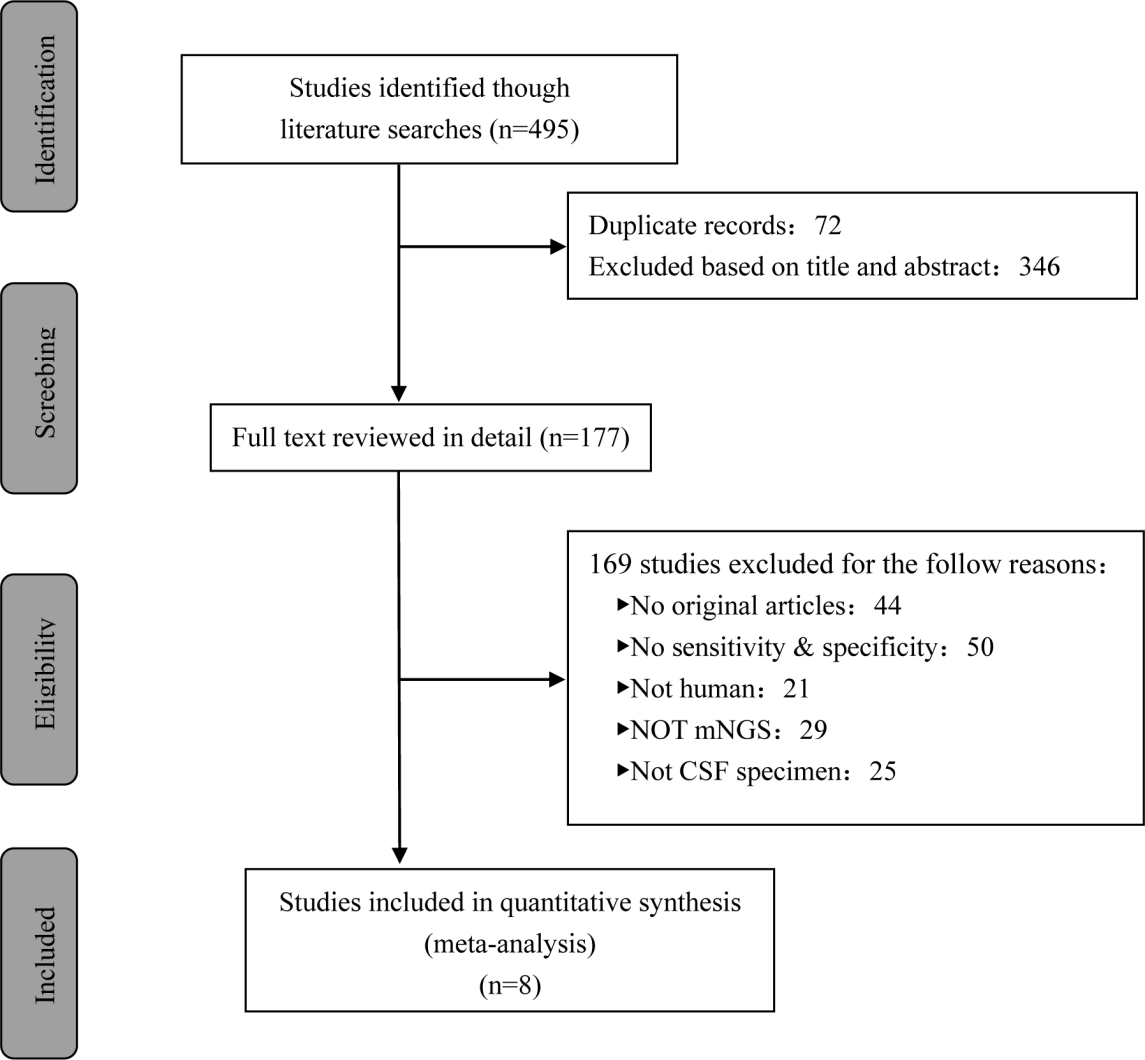
The study selection process flowchart.

A total of 495 candidate articles were retrieved. According to the inclusion criteria, eight studies published between 2019 and 2022 were included in the final meta-analysis (17–24). The selection process is shown in **Figure 1**. A total of 693 participants were included in the eight studies, including 348 patients with TBM and 345 non-TBM controls. Of these studies, three were prospective (18, 19, 21) and five were retrospective (17, 20, 22–24) in design. The patients were randomly selected in three studies (18, 21, 22) and consecutively selected in five studies (17, 19, 20, 23, 24). Fresh CSF samples were used for testing in three studies (20, 22, 24), frozen CSF was used in three studies (17, 19, 21), both fresh and frozen CSF were used in one study (18), and samples were not described in detail in one study (23). All studies used a CRS as a diagnostic reference standard. In two studies, ultrasonication was used (18, 22); in five studies, bead beating was used (17, 19–21, 24); and in one study, the sample pretreatment method was not described in detail (23). The characteristics and relevant data of the included studies are shown in **Tables 1**, **2**.

**Table 1:** Baseline characteristics of the studies that were included.

Study	Sample size(n/N)	Age(years)	Study design	QUADUS score	Specimen condition	Specimen pretreatment	Patient selection
Zhou2019	16/33	49.3/46.3 ^†#^	Prospective	11	Fresh/Frozen	Ultrasonication	Consecutive
Wang2019	23/6	37 ^†^	Retrospective	11	Frozen	Bead beating	Random
Xing2020	44/169	41 (14-76) ^§&^	Prospective	12	Frozen	Bead beating	Random
Yan2020	45/6	34 (19-80)/34.5(24-55) ^¶^	Retrospective	11	Fresh	Bead beating	Random
Sun2021	45/3	29.6 ± 18.1^‡*^	Retrospective	11	Fresh	Ultrasonication	Consecutive
Lin2021	34/16	35.67 ± 6.89 ^‡^	Prospective	12	Frozen	Bead beating	Consecutive
Yu2021	24/13	59/60.5 ^¶^	Retrospective	10	unclear	unclear	Random
Chen2022	117/99	40 (25-53) ^¶^	Retrospective	11	Fresh	Bead beating	Random

n, TBM group.

N, Non TBM group.

^†^ Mean.

^‡^ Mean ± SD.

^§^Mean (range).

^¶^ Median or Median (IQR).

^#^Tuberculosis/Non- tuberculosis group.

^&^ Tuberculous meningitis.

* Extrapulmonary tuberculosis.

**Table 2:** Principal data characteristics of included studies.

Author	Year	Country	Patients	Diagnostic methods (N)	Reference standard	Test result			
						TP	FP	FN	TN
Zhou	2019	China	49	Culture, Xpert	CRS	7	1	9	32
Wang	2019	China	29	Culture (1), AFB (8), PCR (3)	CRS	18	0	5	6
Xing	2020	China	213	Xpert (5), AFB (1)	CRS	12	6	32	163
Yan	2020	China	51	AFB (0), MGIT960 (10), RT-PCR (11), Xpert (18)	CRS	38	0	7	6
Sun	2021	China	48	MGIT960, Xpert	CRS	38	0	7	3
Lin	2021	China	50	MGIT960 (6), FQ-PCR (5), Xpert (13)	CRS	20	0	14	16
Yu	2021	China	37	AFB (2), Culture (4), Xpert (8)	CRS	10	0	14	13
Chen	2022	China	216	Modified Z-N (67), MGIT960 (22), Xpert (65)	CRS	74	0	43	99

AFB, acid fast bacilli.

Z-N, Ziehl-Neelsen staining.

MGITs, mycobacteria growth indicator tubes.

PCR, polymerase chain reaction.

RT-PCR, real-time fluorescence quantitative RCR.

FQ-PCR, Real-time fluorescent quantitative PCR.

TP, true-positive.

FP, false-positive.

FN, false-negative.

TN, true-negative.

CRS, composite reference standard.

### 3.2 Study quality

**Figure 2:**
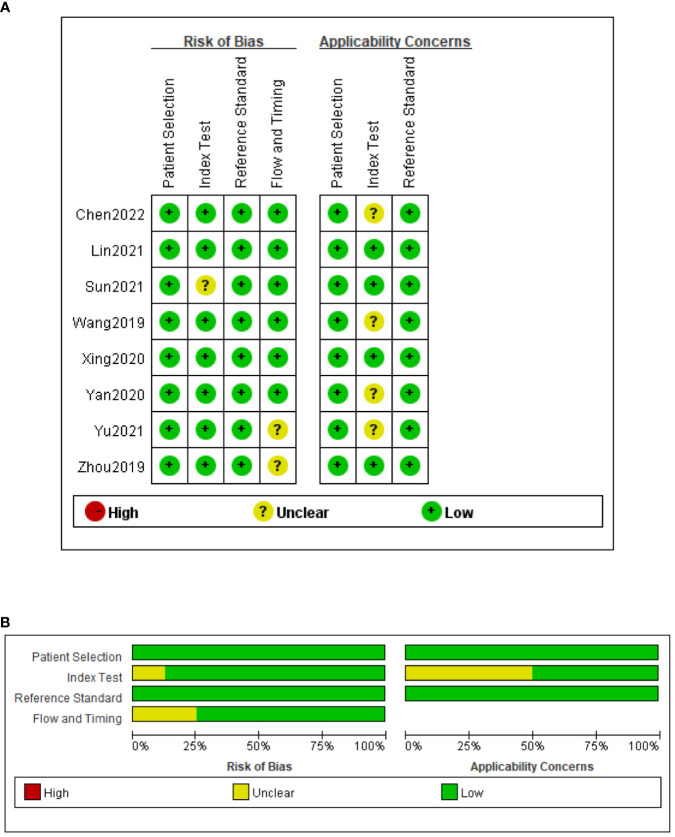
Risk of bias and applicability concerns summary **(A)**; risk of bias and applicability concerns graph **(B)**.

The QUADAS-2 tool was used to assess the quality of all eligible studies in terms of four aspects: patient selection, index test, reference standard, and flow and timing (**Figure 2**). As the selected studies included consecutive or randomly selected patients, the risk of bias in terms of patient selection was assessed as low. The risk of bias in the index test was unclear in one study, and the applicability of the index test was unclear in four studies. All studies used CRS as the reference standard for diagnosing TBM, which could correctly distinguish the target disease and the risk of bias in the reference standard was assessed as low.

### 3.3 Meta-analysis results

#### 3.3.1 Diagnostic accuracy of mNGS for TBM

**Figure 3:**
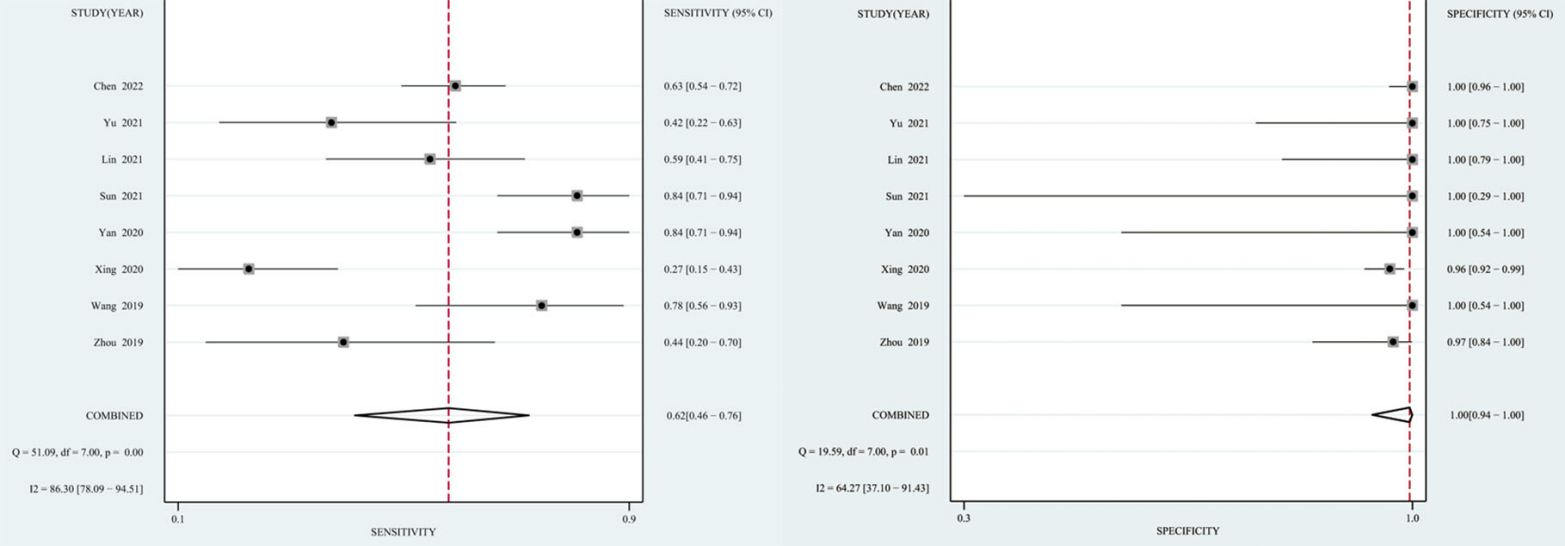
Forest plot of the sensitivity and specificity of pooled testing for mNGS.

**Figure 4:**
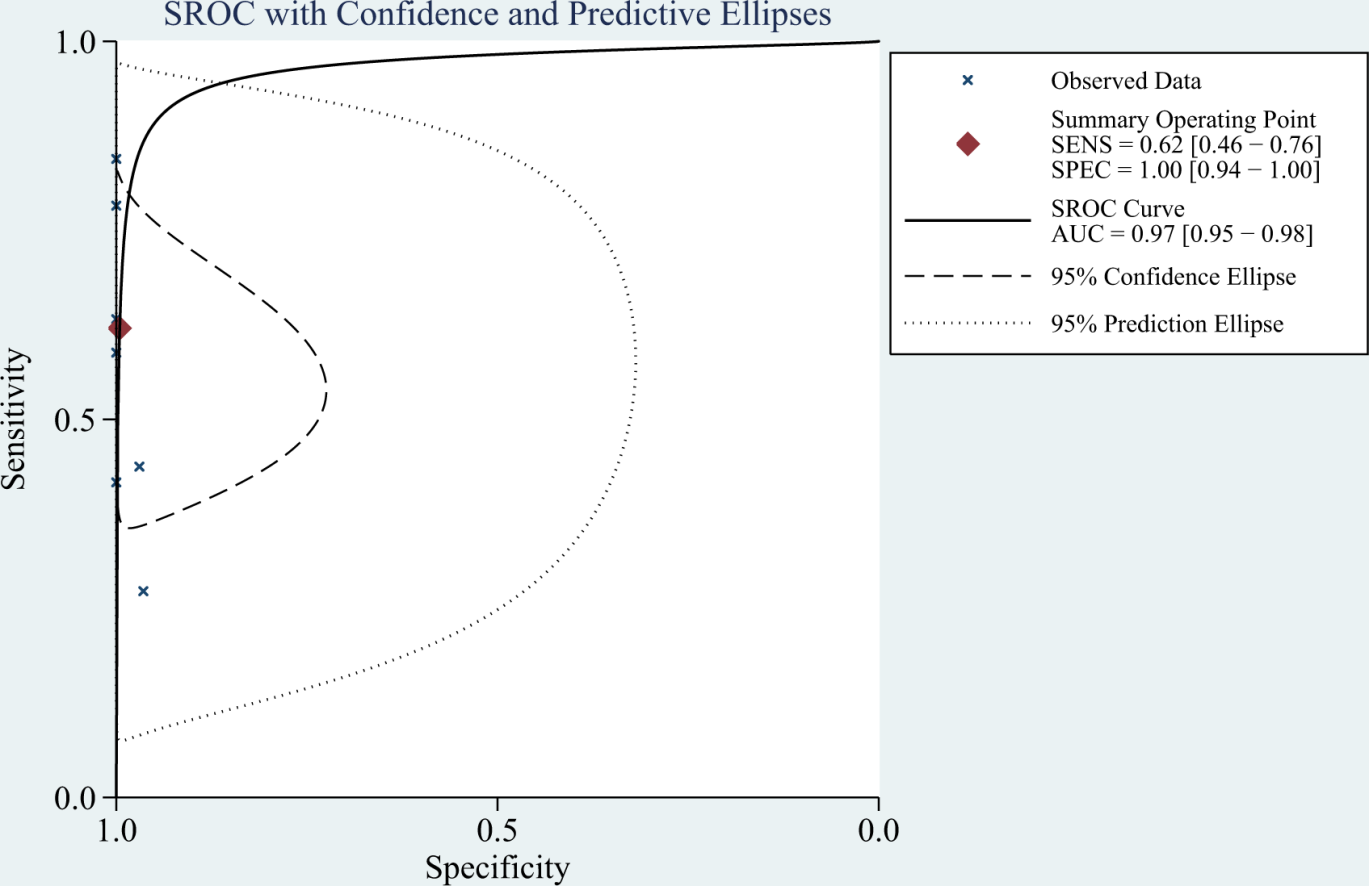
Receiver-operating characteristic curve of mNGS for diagnosing TBM.

The *I* ^2^ value and Cochrane Q test results showed significant heterogeneity between studies in terms of sensitivity (*I* ^2 =^ 86.3%, *P* < 0.01), specificity (*I* ^2 =^ 64.27%, *P* = 0.01), and DOR (*I* ^2 =^ 100%, *P* < 0.01); therefore the random-effects model was used in the meta-analysis. The pooled sensitivity, specificity, PLR, NLR, DOR and AUC of the SROC of mNGS for diagnosing TBM was 62% (95% CI: 0.46–0.76), 99% (95% CI: 0.94–1.00), 139.08 (95% CI: 8.54–2266), 0.38 (95% CI: 0.25–0.58), 364.89 (95% CI: 18.39–7239) and 0.97 (95% CI: 0.95–0.98), respectively (**Figures 3**, **4**).

#### 3.3.2 Publication bias analysis

**Figure 5:**
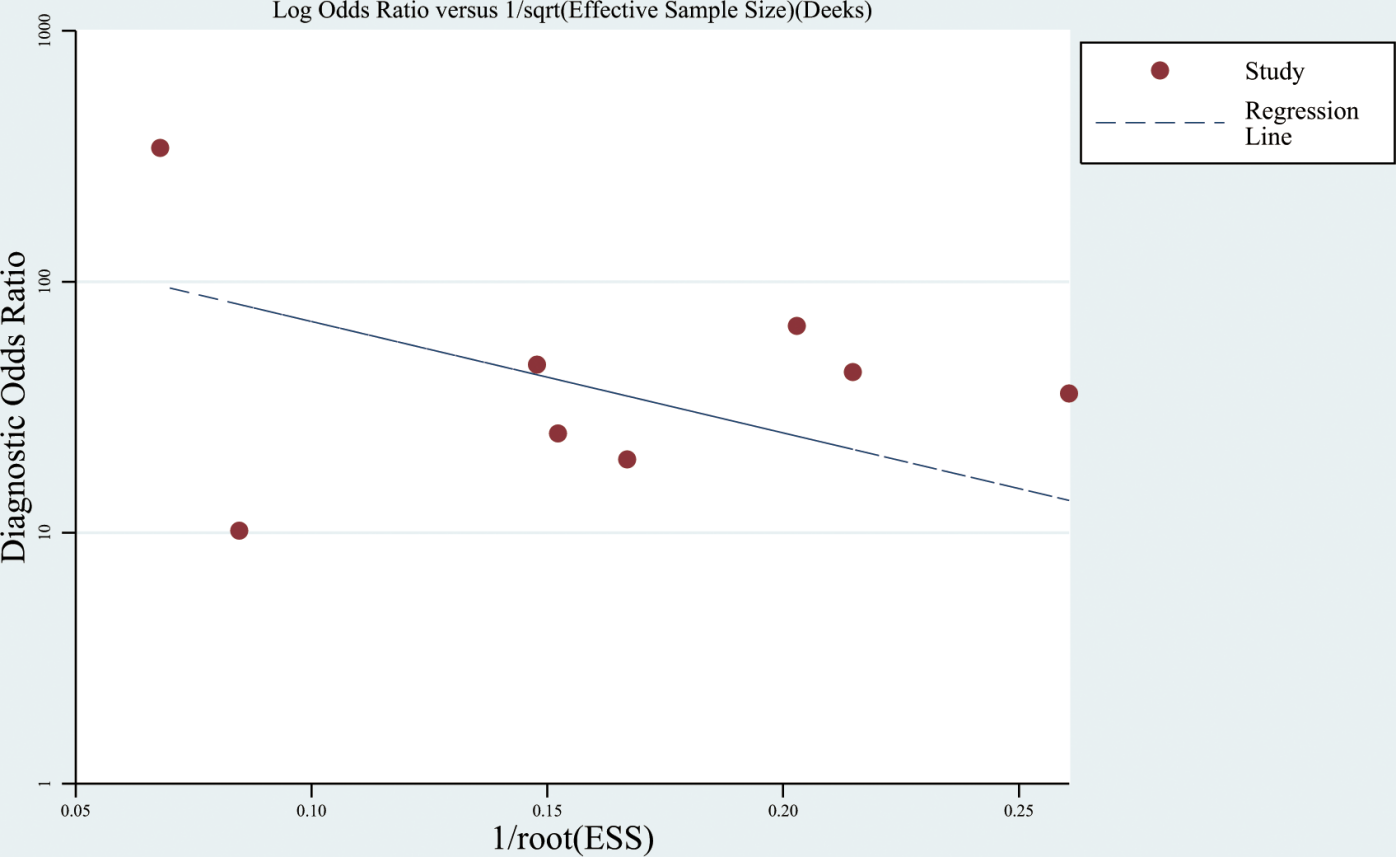
Deeks’ funnel plot of the included studies.

The Deeks’ funnel plot showed no significant publication bias (*P* = 0.372) (**Figure 5**).

#### 3.3.3 Post-test probability of TBM

**Figure 6:**
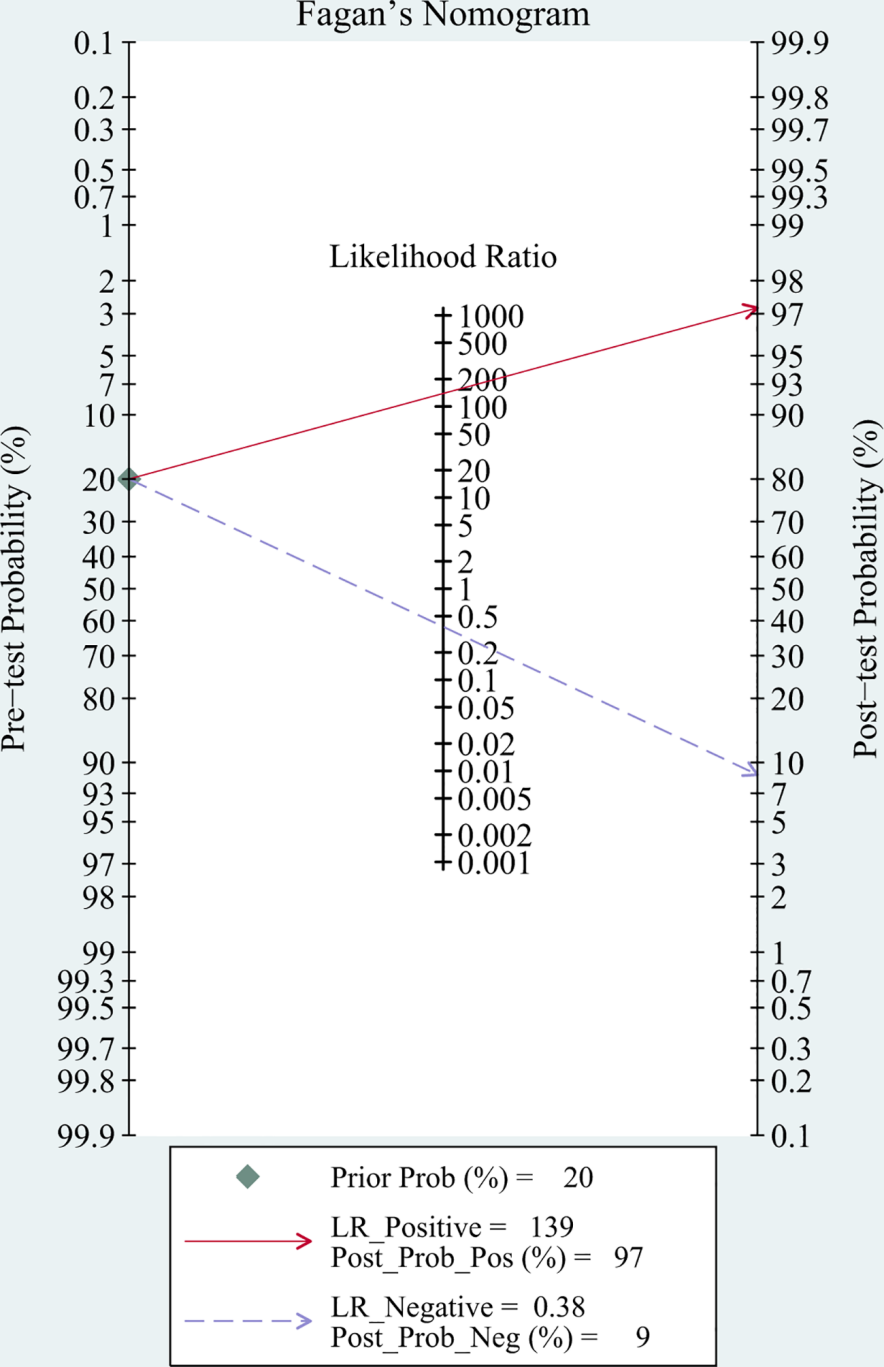
Post-test probabilities of TBM for CSF mNGS.

Fagan’s nomogram showed that the pre-test probability of TBM was 20%, and the post-test probability increased to 97% if a patient tested positive using mNGS. If the mNGS test result was negative, the likelihood of the patient having TBM decreased to 9% (**Figure 6**).

## 4 Discussion

Identification of MTB in the CSF is the key to diagnosing TBM. As MTB is an intracellular bacterium, the pathogen content in CSF is extremely low, and its concentration rarely exceeds 100–1000 colonies per mL(7), whereas acid-fast staining requires that the MTB load in CSF be >10,000 organisms, resulting in a positive result acquired by acid-fast staining for TBM diagnosis of only 10–40% (25, 26). Even though some studies have shown that the modified acid-fast staining method improves the sensitivity for diagnosing TBM (27, 28), other studies have shown that the sensitivity of this method is inadequate for clinical application (25). Compared with acid-fast staining, CSF culture improved the probability of MTB detection; the sensitivity reached 50–70% (26, 29), and the drug sensitivity test could be carried out simultaneously (30). Nevertheless, this method is time-consuming and often causes delays that make the early diagnosis of TBM difficult. Additionally, MTB culture should be performed in a biosafety level III laboratory, which indirectly increases the cost of testing samples (5) (**Table 3**).

**Table 3:** Comparison of mNGS and conventional/novel diagnostic tests for tuberculous meningitis performed on CSF specimens.

Tests for TBM diagnosis		Sensitivity (%)	Specificity (%)	Key points	References
**Ziehl-Neelsen staining**		10-40	100	Sensitivity substantially improved by increasing the volume of CSF(>6mL), prolonging slide examination(>30min) and examining multiple specimens.	Bahr et al. (26) Heemskerk et al. (25)
**Mycobacterial culture** **•** Solid culture **•** Liquid culture		50-70 (L-J culture) Improve the MTB detection rate up to 10% than solid format (Liquid media)	100	High requirements (biosafety Level III) on the laboratory. Long time to result (3-5 weeks for solid medium; within 2 weeks for liquid medium), and impossible to provide information for clinical diagnosis during the acute phase of TBM.	Thwaites et al. (30) Bahr et al. (26) Ahlawat et al. (29)
**Xpert MTB/RIF Ultra**		44-93	94-100	A potential gamechanger. It is a rule-in testing, but not a confidently rule-out test. Requires further evaluation.	Wang et al. (31) Cresswell et al. (32)
**ADA**		60-90	80-90	Provide variable results. Fail to differentiate between purulent meningitis and TBM.	Pormohammad et al. (33) Ekermans et al. (34)
**CSF IGRAS**		79-81	89-95	Cut-off range and incubation cell numbers across the studies were inconsistent. Fail to differentiate between active and latent TB.	Wen et al. (35) Lan et al. (36)
**mNGS**		62% (95% CI: 0.46–0.76)	99% (95% CI: 0.94–1.00)	Prone to contamination. Its specificity is extremely high, but its sensitivity is moderate. Requires a large volume of CSF, relatively long detection time and high cost. Very few studies, small subject numbers.	Present

L-J, Lowenstein-Jensen.

mNGS, metagenomic next-generation sequencing.

CSF, cerebrospinal fluid.

ADA, adenosine deaminase.

IGRA, interferon-gamma release assay.

TB, tuberculosis.

TBM is a type of paucibacillary EPTB (7), which limits the application of traditional etiological detection methods. With the development of modern precision medicine technology, molecular diagnostics has been widely paid attention to and applied. Xpert MTB/RIF Ultra (Xpert Ultra) is a novel NAAT that can be used to detect MTB genes and rifampicin resistance in 1.5–2 hours (32, 37). Xpert Ultra is rapid and highly automated, with higher biosafety than smear microscopy and lower cross-contamination risk than culture (32, 38, 39). In 2017, the World Health Organization recommended Xpert Ultra as the initial test for TBM diagnosis (40). Although Xpert Ultra has advantages over other classical microbiological methods (31), its sensitivity is still unsatisfactory (41, 42), and a negative result cannot rule out TBM (32, 43). However, its high requirements for sample size and bacterial load, and unsatisfactory performance in EPTB, HIV coinfection, and children limit its application in clinical practice (32, 40) (**Table 3**).

Adenosine deaminase (ADA) detection has the advantages of being simple, rapid, and stable, which makes it useful as a reference test for the diagnosis of TBM. A meta-analysis showed that ADA had a sensitivity of 89% and specificity of 91% for diagnosing TBM (33). However, there is no clear cutoff for the diagnosis of TBM, and it is difficult to distinguish TBM from suppurative meningitis and viral meningitis (33, 34). Clinical manifestations and other relevant diagnostic tests are required for the diagnosis of TBM. Interferon-gamma release assays (IGRAs) are used to detect the presence of MTB infection by measuring the amount of interferon (IFN)-gamma released by T cells after stimulation with MTB-specific antigens and the number of T cells releasing IFN-gamma (44). A meta-analysis showed that the overall sensitivity and specificity of IGRA 74% and 79%, respectively, in blood, and 78% and 95%, respectively, in CSF, suggesting moderate accuracy in the diagnosis of TBM (35, 36). However, IGRA is not effective at distinguishing between latent and active TB (45, 46). CSF IGRAs also has the disadvantages of requiring a large volume of CSF (> 4 mL) and an uncertain critical value (32, 47–49) (**Table 3**).

More recently, the CSF mNGS has been gradually adopted for the diagnosis of infectious diseases of the CNS (13, 50–52). Although research on the diagnosis of TBM is still in its infancy, some progress has been made. In 2020, Yu et al. conducted the first meta-analysis of mNGS diagnosis of TBM (a total of four studies were included) and found that the sensitivity of mNGS detection was 62%, while the specificity was as high as 98% (53). However, owing to the small number of studies included in this meta-analysis (<5 studies) and the small sample size of the included studies (342 patients), the results and conclusions should be interpreted with caution. Eight studies were included in this meta-analysis. Comprehensive analysis showed that the pooled sensitivity and specificity of mNGS for the diagnosis of TBM was 62% and 99% (i.e., the missed diagnosis and misdiagnosis rates were 38% and 1%, respectively), and the pooled DOR and SROC areas were 364.89 and 0.97, respectively. The results of this study are similar to the sensitivity and specificity of mNGS obtained by Yu et al., which further validates the diagnostic efficacy of mNGS. Compared with Yu et al.’s study, this study included more studies and sample sizes, and established more detailed and strict inclusion and exclusion criteria. By integrating all relevant studies, this meta-analysis could more accurately evaluate the diagnostic efficacy of mNGS.

In 2020, a prospective multicenter randomized controlled study conducted by Donovan et al. found that the sensitivities of Xpert Ultra and Xpert in diagnosing TBM were 47.2% and 39.6%, respectively, with a specificity of 100.0% (43). In the same year, a meta-analysis of 14 articles found that Xpert had a pooled sensitivity of 63% and specificity of 98.1% for diagnosing TBM (54). In 2021, Shen et al. conducted a meta-analysis using Xpert Ultra and Xpert for the diagnosis of TBM, and the results suggested that the sensitivity of Xpert Ultra (68%) was higher than that of Xpert (37%), but the specificity of both was up to 100% (42). Comprehensive analysis showed that mNGS had no significant advantage in diagnosing TBM sensitivity compared to Xpert Ultra and Xpert. However, the specificity of all three methods was >95%, indicating a low misdiagnosis rate. According to relevant studies, mNGS has moderate sensitivity, good specificity, and high accuracy for diagnosing TBM. In addition, mNGS can detect almost all pathogens, including viruses, bacteria, fungi, and parasites, is rapid, and has a high throughput (11, 24, 55) (**Table 3**).

The emergence of mNGS provides a novel approach to the diagnosis of TBM; however, many problems and challenges remain. First, the operation process of mNGS is complicated and it requires high levels of laboratory infrastructure and technical proficiency of operators (55). Second, the test results are subject to many factors, and their interpretation depends on the professional knowledge and clinical experience of the clinician (11). Third, owing to the relatively high testing cost of mNGS, its clinical use is limited, particularly in low- and middle-income countries (11). Fourth, the etiological database is inadequate. Finally, mNGS cannot directly detect drug sensitivity, which limits its use as a guide to antituberculosis therapy (56). The present analysis has several limitations: First, the search scope was limited to published literature, and unpublished studies and gray literature may have been missed, so potential publication bias cannot be ruled out. Second, microbiological confirmation was not achieved in all TBM cases, which may have affected the reliability of the results. Third, among the eight eligible studies, all were conducted in China, and most involved adult patients. Studies on patients in low-burden areas and children are lacking. Four, the duration of anti-tuberculosis treatment in the included literature is unknown; and anti-tuberculosis treatment before mNGS detection may affect their diagnostic sensitivity, which will also cause heterogeneity among the included studies. Finally, the number of studies and the amount of clinical data were relatively limited, and the sample conditions and pretreatment methods differed among the studies.

## 5 Conclusion

In summary, current evidence shows that mNGS has good specificity for the diagnosis of TBM; however, its sensitivity is moderate. The high requirements for laboratory infrastructure and high cost, make mNGS unsuitable for use as an initial test for TBM in the short term. However, it should be used as an effective pathogen-screening method to diagnose patients with negative results to microbiological tests, failure of empirical therapy, and critical illness. Owing to the limited quality and quantity of the included studies, these conclusions need to be interpreted with caution. Additional high-quality prospective, large, multicenter studies are required to confirm the diagnostic value of mNGS for TBM in a more comprehensive, systematic, scientific, and objective manner.

## Author contributions

Z-BX and AW had the original idea of this study. E-LL and W-FC designed the research. The article was written by AW and Z-BX. S-ML and Y-LZ contributed to the data searches and study selection. C-QL and FH analyzed data and created the tables and figures. All authors contributed to the article and approved the submitted version.
